# Sequencing and analysis of the complete mitochondrial genome of *Culex tritaeniorhynchus* (Dipera: Culicidae) in China

**DOI:** 10.1080/23802359.2016.1168719

**Published:** 2016-06-20

**Authors:** Hongliang Chu, Zhiming Wu, Hengduan Zhang, Chunxiao Li, Gang Wang, Minghao Zhou, Tongyan Zhao

**Affiliations:** a State Key Laboratory of Pathogen and Biosecurity, Beijing Institute of Microbiology and Epidemiology, Fengtai District, Beijing, China;; b Department of Disinfection and Vector Control, Jiangsu Provincial Center for Disease Control and Prevention, Gulou District, Nanjing, China;; c Zhejiang Entry-Exit Inspection, Quarantine Bureau of the PR China, Hangzhou, China

**Keywords:** *Culex tritaeniorhynchus*, mitochondrial genome, phylogenetic analysis

## Abstract

The complete sequence of the mitochondrial genome of the *Culex tritaeniorhynchus* (Diptera: Culicidae) is presented using traditional Sanger sequencing. Its mitogenome are 15,123 bp in length, consisting of 13 protein-coding genes, 2 ribosomal RNA genes (rRNA) and 22 transfer RNA (tRNA) genes. The percent of A + T of the mitogenome is 77.73%. Most mitochondrial genes are encoded on the heavy strand, except for *NAD5*, *NaD4*, *NAD4L*, *NAD1*, two rRNA and nine tRNA genes, which are encoded on the light strand. The results of phylogenetic analyzes showed that the *Cx. tritaeniorhynchus* has close relationship with the species of genus *Culex*.

*Culex tritaeniorhynchus* (Diptera: Culicidae) is the most important vector of Japanese Encephalitis virus. It even can be superinfected with different viruses (Kuwata et al. 2015). *ND4* and *ND5* sequences of mosquito mitochondrion can provide evidence of population genetic structure and gene isolation by distance (Gorrochotegui-Escalante et al. 2000; Birungi & Munstermann 2002; Szalanski et al. 2006; Venkatesan et al. 2007). Cytochrome C oxidase I (COI) gene in mosquito mitochondrion is a very important target for identification DNA barcodes (Kumar et al. 2007; Wang et al. 2012; Ondrejicka et al. 2014). We first determined the mitochondrial genome of *Cx. tritaeniorhynchus* (GenBank accession no. KT852976). The sample were collected in Nanjing, China (geographic location: 118.84159°E, 32.01337°N) in August 2014. The collected specimen is stored in the Medical Insect Collection of Beijing Institute of Microbiology and Epidemiology, and its accession number is ME-Z-III-26-08.

The complete mitogenome of this individual is 15,123 bp in length, and consists of 13 protein-coding genes, two ribosomal RNA genes (rRNA) and 22 transfer RNA (tRNA) genes. The percent of A + T of the mitogenome is 77.73%. Mitochondrial DNA is circular double strands including heavy strand and light strand. Most mitochondrial genes are encoded on the heavy strand, except for *NAD5*, *NAD4*, *NAD4L*, *NAD1*, two rRNA and nine tRNA genes, which are encoded on the light strand. There are six mitochondrial protein-coding genes share the start codon ATG which are *COX2*, *ATP6*, *COX3*, *NAD4*, *NAD4L* and *COB*. The *ATP8, NAD1, NAD6* and *NAD6* are initiated with the start codon ATA. Both *NAD2* and *NAD5* are initiated with TCG. And the *COX1* is initiated by TCG. The *COX1*, *COX2* and *NAD6* are terminated by TAA and AAA, respectively. The remaining 12 protein-coding genes end with the typical termination codon TAA. It is expected that this complete mitogenome could be used to investigate the phylogenetic relationship of *Cx. tritaeniorhynchus* in Culicidae and provide other more information.

In order to explore the evolution situation of *Cx. tritaeniorhynchus* in Diptera, we analyze the molecular phylogenetics of Diptera using the complete mitochondrial genome sequence of 18 species which are downloaded from the GenBank (Figure 1). The 18 species come from three families: Culicidae, Muscidae and Drosophilidae.

**Figure 1.**
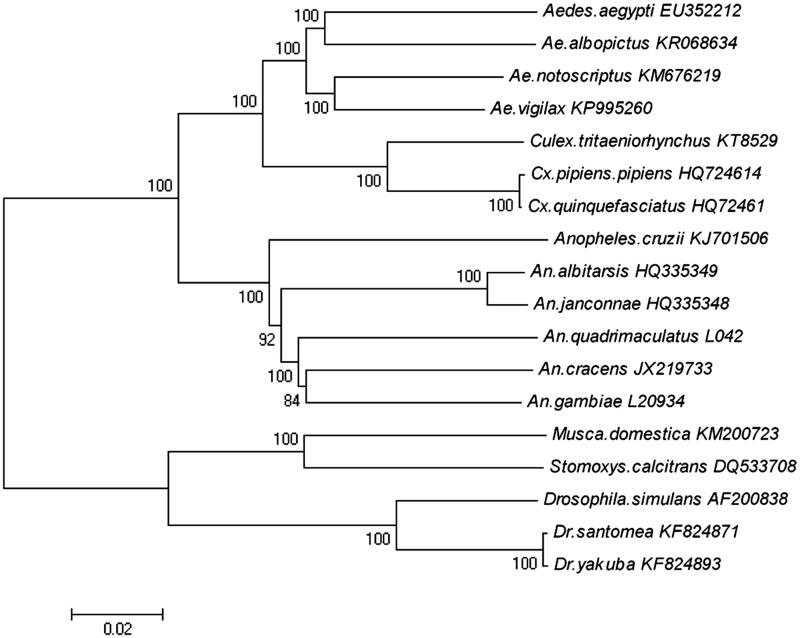
Phylogenetic tree generated using the neighbour-joining method based on complete mitochondrial genomes. *Aedes aegypti* (EU352212), *Ae. albopictus* (KR068634), *Ae. notoscriptus* (KM676219), *Ae. vigilax* (KP995260), *Culex tritaeniorhynchus* (KT852976), *Cx. pipiens pipiens* (HQ724614), *Cx. quinquefasciatus* (HQ724617), *Anopheles alitarsis* (HQ335349), *An. janconnae* (HQ335348), *An. quadrimaculatus* (L04272), *An. cracens* (JX219733), *An. gambiae* (L20934), *Musca domestica* (KM200723), *Stomoxys calcitrans* (DQ533708), *Drosophila simulans* (AF200838), *Dr. santomea* (KF824871) and *Dr. yakuba* (KF824893).

The mitochondrial genome was analyzed with neighbour-joining method, and the phylogenetic tree obtained is shown in Figure 1. It shows that the three families in Diptera are phyletic lineages. In the three families, Culicidae comprised *Aedes aegypti* (EU352212), *Ae. albopictus* (KR068634), *Ae. notoscriptus* (KM676219), *Ae. vigilax* (KP995260), *Culex tritaeniorhynchus* (KT852976), *Cx. pipiens pipiens* (HQ724614), *Cx. quinquefasciatus* (HQ724617), *Anopheles alitarsis* (HQ335349), *An. janconnae* (HQ335348), *An. quadrimaculatus* (L04272), *An. cracens* (JX219733), *An. gambiae* (L20934); Mucidae comprised *Musca domestica* (KM200723); *Stomoxys calcitrans* (DQ533708). Drosophilidae comprised *Drosophila simulans* (AF200838), *Dr. santomea* (KF824871), *Dr. yakuba* (KF824893).
